# Reinvestigation of the crystal structure of UF_6_ by single-crystal X-ray diffraction

**DOI:** 10.1107/S2414314626008898

**Published:** 2026-08-28

**Authors:** Björn-Niclas Koch, Florian Kraus

**Affiliations:** a Anorganische Chemie, Fluorchemie, Universität Bonn, Gerhard-Domagk-Str. 1, 53121 Bonn, Germany; Vienna University of Technology, Austria

**Keywords:** crystal structure, uranium hexa­fluoride, double hexa­gonal packing, actinoid fluoride, redetermination

## Abstract

Single-crystals of hexa­fluoridouranium(VI), UF_6_, were obtained by sublimation. A re-refinement of the crystal structure using low-temperature (100 K) single-crystal X-ray data was carried out considering all *U^ij^* terms of the displacement parameters of the U and F atoms, leading overall to a significantly improved structural model. UF_6_ crystallizes in the ortho­rhom­bic space group *Pnma* with the F atoms forming a double hexa­gonal closest packing of *ABAC* stacking with U atoms in one-sixth of the voids. The UF_6_ mol­ecules deviate only slightly from octa­hedral point-group symmetry.

## Abstract 2

Re-refinement of the crystal structure of UF_6_ using single-crystal X-ray diffraction data at 100 K allowed to obtain a more precise structural model in comparison with previous studies.

## Graphical abstract

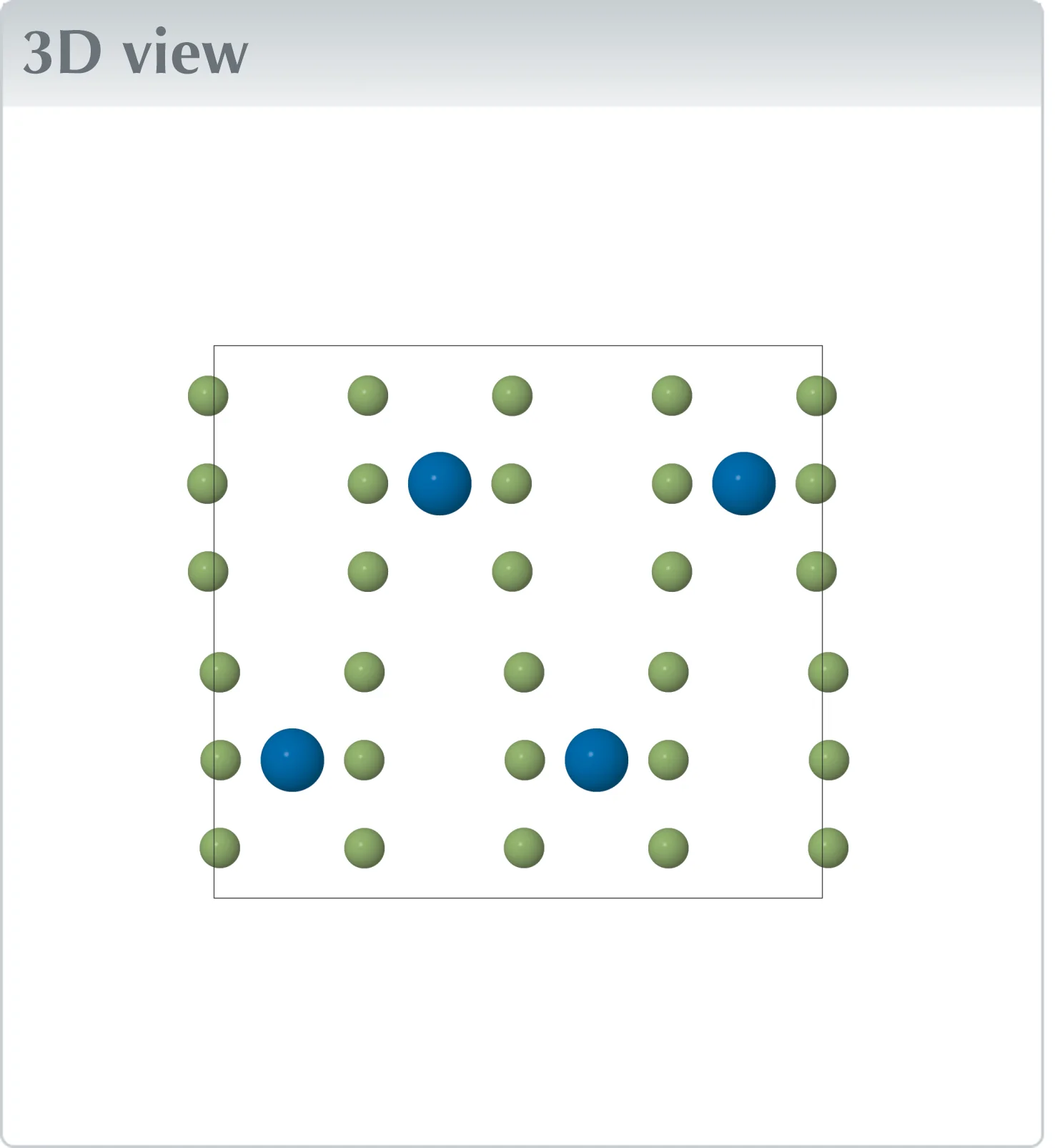

## Structure description

Uranium hexa­fluoride is likely the most important material in the nuclear energy sector for isotope enrichment of the fissile isotope ^235^U. UF_6_ was probably first synthesized by Henri Moissan (1900 ▸) by reacting fluorine gas with uranium metal. However, at that time he was unable to further characterize the resulting white smoke. Later, Otto Ruff and coworkers succeeded in obtaining UF_6_ and describing its properties (Ruff, 1909 ▸; Ruff & Heinzelmann, 1911 ▸). Ruff reported shiny, colorless, crystals with monoclinic symmetry without giving further details (Ruff, 1909 ▸). Reports on the crystal structure of UF_6_ appeared well after the Manhattan Project. The first one, a single-crystal X-ray diffraction study of a crystal mounted in a glass capillary (Hoard & Stroupe, 1958 ▸) led to lattice parameters *a* = 9.900 (2), *b* = 8.962 (2), *c* = 5.207 (2) Å, *V* = 462.0 Å^3^ at *T* = 298 K in space group *Pnma* (no *R* values reported). In 1973, the space group and structure model were confirmed by powder neutron diffraction at 294 K using the previously reported lattice parameters (Taylor *et al.*, 1973 ▸) without giving *R* values. In 1975, another structural study of UF_6_ at 193 and 293 K by powder neutron diffraction was carried out (Taylor & Wilson, 1975 ▸), confirming that it has ortho­rhom­bic symmetry at both temperatures [*a* = 9.843 (11), *b* = 8.920 (10), *c* = 5.173 (6) Å, *V* = 454.2 (8) Å^3^ at *T* = 193 K, *R* = Σ(|*I*_o_ – *I*_c_|)/Σ*I*_o_ = 0.081; *a* = 9.924 (10), *b* = 8.954 (9), *c* = 5.198 (5) Å, *V* = 461.9 (8) Å^3^ at *T* = 293 K, *R* = Σ(|*I*_o_ – *I*_c_|)/Σ*I*_o_ = 0.133]. Levy *et al.* (1976 ▸) reported a single-crystal neutron diffraction study at 293 K with *a* = 9.92 (5), *b* = 8.97 (5), *c* = 5.22 (3) Å (*V* not reported), and gave an *R* value of 0.041. The latest report refers to a powder neutron diffraction study at 77 K, which led to lattice parameters *a* = 9.654 (3), *b* = 8.776 (4), *c* = 5.084 (3) Å, *V* = 430.7 (3) Å^3^ and an *R* = Σ(|*y*_o_ – *y*_c_|)/Σ*y*_o_ value (*y*_o_ and *y*_c_ are the background-corrected pattern intensities) of 0.069 (Levy *et al.*, 1983 ▸).

As all of these structure refinements, with the exception of the single-crystal neutron diffraction study by Levy *et al.* (1976 ▸), used isotropic displacement parameters for all atoms, we reinvestigated the crystal structure of UF_6_ based on single-crystal X-ray diffraction data at 100 K. Refinement of all *U^ij^* terms of the displacement parameters of the U and the four F atoms allowed for a more precise structural model.

**Figure 1:**
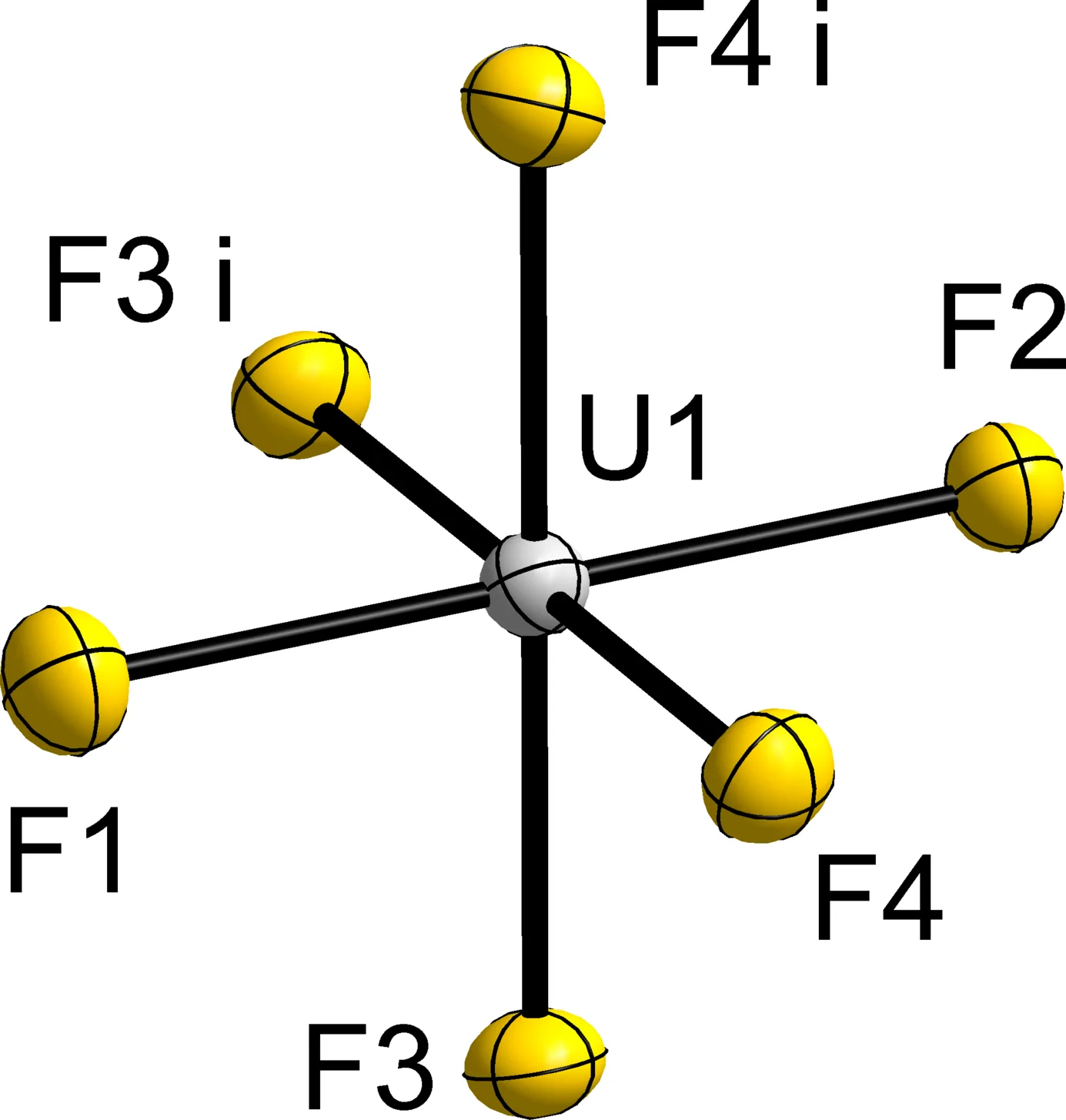
UF_6_ mol­ecule in the solid state. Atoms are shown with anisotropic displacement ellipsoids at the 70% probability level; the symmetry code refers to Table 1 ▸.

The U atom (multiplicity 4, Wyckoff letter *c*, site symmetry .*m*.) is surrounded by six F atoms (F1 to F4) in a slightly distorted octa­hedral arrangement (Fig. 1 ▸, Table 1 ▸). The F1 and the F2 atoms likewise reside on a mirror plane (4 *c*, .*m*.), while the F3 and F4 atoms occupy a general position (8 *d*, 1). In the previous studies, the U—F bond lengths were reported to range from 1.88 (2) to 2.28 (4) Å with F—U—F angles from 88.0 (17) to 92.9 (17)° (Taylor *et al.*, 1973 ▸), from 1.95 (1) to 2.03 (2) Å at 193 K with angles from 86.4 (7) to 92.2 (6)°, from 1.86 (3) to 1.99 (2) Å with angles from 83.7 (9) to 95.1 (8)° (Taylor & Wilson, 1975 ▸), from 1.992 (3) to 2.004 (4) Å with angles from 89.42 (17) to 90.20 (11)° (Levy *et al.*, 1976 ▸), and as mean bond lengths of 2.023 (6) Å (77 K), 1.983 (6) Å (193 K), and 1.995 (2) Å (293 K) without reporting the bond angles (Levy *et al.*, 1983 ▸). It is inter­esting to note that the longest bond lengths were observed at the lowest temperature, underlining the need for a redetermination of the crystal structure. The U—F bond lengths determined in the present work are much more uniform, and the F—U—F bond angles are closer to the ideal values of 90 and 180° than determined in previous studies (Table 1 ▸). The deviations of the UF_6_ mol­ecule from *O_h_* symmetry are thus small; its crystallographically imposed point symmetry is *C_s_* (*m*).

The unit-cell parameters determined in this study (Table 2 ▸) are in good agreement with those given above, especially with those determined at 77 K (Levy *et al.*, 1983 ▸) which are a little smaller, as expected.

**Figure 2:**
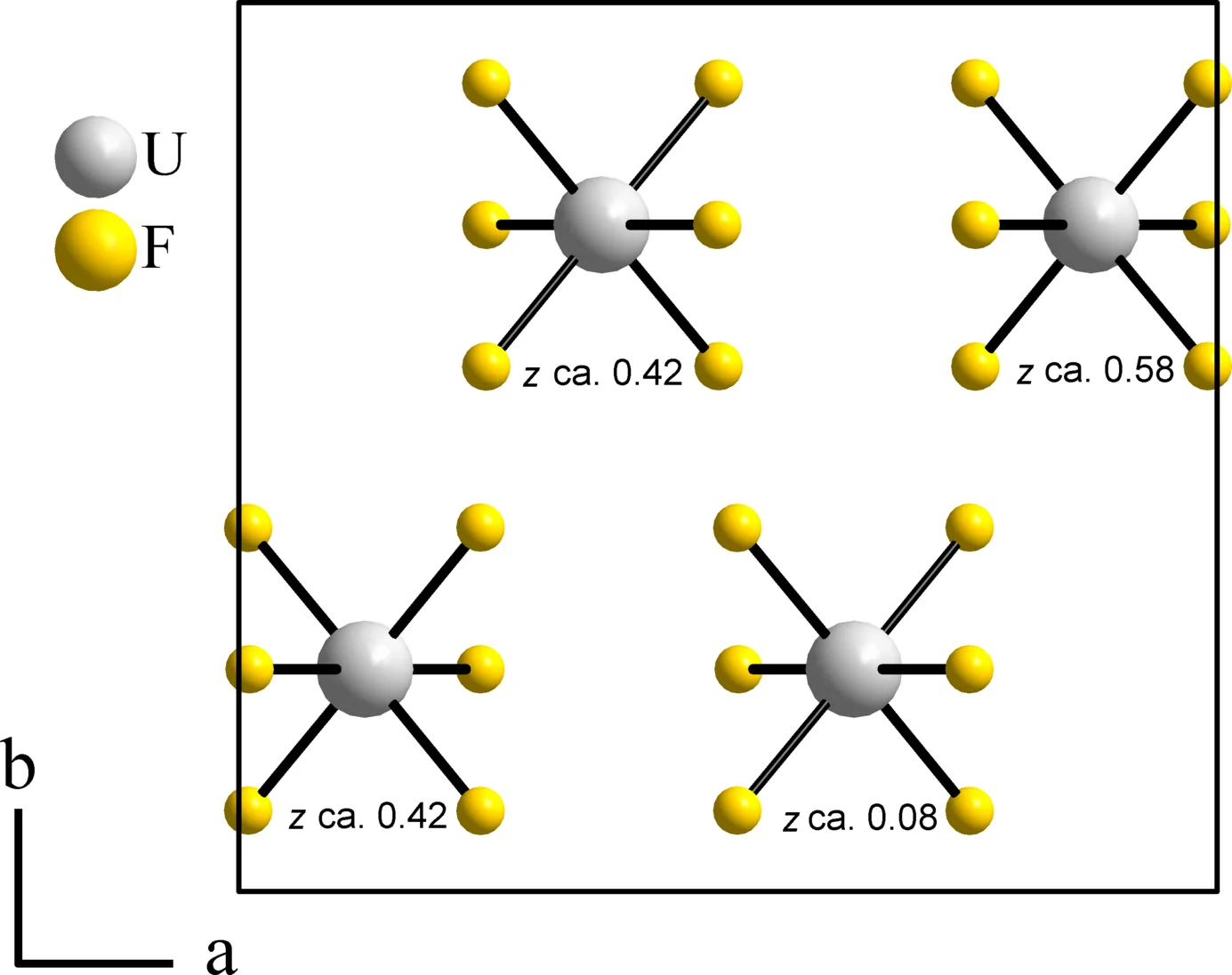
Crystal structure of UF_6_ viewed along the *c* axis. Atoms are drawn as spheres with arbitrary radii. The *z* coordinates of the U atoms are indicated.

In the crystal structure, a single UF_6_ mol­ecule is surrounded by twelve others in the shape of a distorted anti­cubocta­hedron if U⋯U distances from 5.0854 (4) to 5.1402 (4) Å are considered. Thus, the U atoms adopt a distorted arrangement similar to the Mg atoms in the Mg structure type. In a more detailed description, the U atoms occupy one-sixth of the octa­hedral voids of hexa­gonally close-packed layers of F atoms. The layers are parallel to the *bc* plane, with a stacking sequence along the *a-*axis direction of *ABAC*, representing a double hexa­gonal packing, *hc*. The close-packing of F atoms is only slightly distorted, as evidenced by the F⋯F distances. Those within a UF_6_ mol­ecule range from 2.791 (6) to 2.808 (4) Å, while the inter­molecular distances are only slightly larger in the range from 2.900 (6) to 3.119 (6) Å. The crystal structure of UF_6_ is shown in Fig. 2 ▸.

## Synthesis and crystallization

UF_6_ of natural isotope ratio was synthesized according to a literature procedure (Chemnitz *et al.*, 2021 ▸), and single crystals were obtained by slow sublimation at room temperature.

## Refinement

Details of the data collection and structure refinement are given in Table 2 ▸.

## Supplementary Material

Crystal structure: contains datablock(s) I. DOI: 10.1107/S2414314626008898/wm4259sup1.cif

Structure factors: contains datablock(s) I. DOI: 10.1107/S2414314626008898/wm4259Isup2.hkl

CCDC reference: 2580701

Additional supporting information:  crystallographic information; 3D view; checkCIF report

## Figures and Tables

**Table 1:** Selected geometric parameters (Å, °)

U1—F1	1.983 (4)	U1—F3^i^	1.984 (3)
U1—F2	1.982 (4)	U1—F4	1.975 (3)
U1—F3	1.984 (3)	U1—F4^i^	1.975 (3)
			
F1—U1—F3	90.12 (11)	F4^i^—U1—F2	89.92 (11)
F2—U1—F1	179.95 (14)	F4—U1—F2	89.92 (11)
F2—U1—F3	89.91 (11)	F4—U1—F3	90.20 (13)
F4^i^—U1—F1	90.05 (11)	F4—U1—F3^i^	179.56 (11)

Symmetry code: (i) 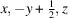.

**Table 2:** Experimental details

Crystal data	
Chemical formula	UF_6_
*M*_r_	352.02
Crystal system, space group	Orthorhombic, *Pnma*
Temperature (K)	100
*a*, *b*, *c* (Å)	9.6612 (8), 8.7786 (7), 5.0854 (4)
*V* (Å^3^)	431.30 (6)
*Z*	4
Radiation type	Mo *K*α
μ (mm^−1^)	37.66
Crystal size (mm)	0.3 × 0.2 × 0.1

Data collection	
Diffractometer	Stoe *IPDS* II
Absorption correction	Multi-scan (*LANA*; Koziskova *et al.*, 2016 ▸)
*T*_min_, *T*_max_	0.0002, 0.001
No. of measured, independent and observed [*I* > 2σ(*I*)] reflections	6472, 784, 681
*R*_int_	0.042
(sin θ/λ)_max_ (Å^−1^)	0.743

Refinement	
*R*[*F*^2^ > 2σ(*F*^2^)], *wR*(*F*^2^), *S*	0.022, 0.054, 1.03
No. of reflections	784
No. of parameters	38
Δρ_max_, Δρ_min_ (e Å^−3^)	2.37, −1.68

Computer programs: *X-AREA* (Folkers-Karlsson *et al.*, 2026 ▸), *SHELXT* (Sheldrick, 2015*a* ▸), *SHELXL* (Sheldrick, 2015*b* ▸), *DIAMOND* (Brandenburg, 2022 ▸), *publCIF* (Westrip, 2010 ▸) and *OLEX2* (Dolomanov *et al.*, 2009 ▸).

## full crystallographic data

### Hexafluoridouranium(VI) Crystal data

**Table:**

UF_6_	*D*_x_ = 5.421 Mg m^−^^3^
*M**_r_* = 352.02	Mo *K*α radiation, λ = 0.71073 Å
Orthorhombic, *P**n**m**a*	Cell parameters from 9312 reflections
*a* = 9.6612 (8) Å	θ = 4.0–32.3°
*b* = 8.7786 (7) Å	µ = 37.66 mm^−^^1^
*c* = 5.0854 (4) Å	*T* = 100 K
*V* = 431.30 (6) Å^3^	Plate, clear colourless
*Z* = 4	0.3 × 0.2 × 0.1 mm
*F*(000) = 584	

### Hexafluoridouranium(VI) Data collection

**Table:**

Stoe IPDS II diffractometer	784 independent reflections
Radiation source: sealed X-ray tube, 12 x 0.4 mm long-fine focus, X-ray tube	681 reflections with *I* > 2σ(*I*)
Planar graphite monochromator	*R*_int_ = 0.042
Detector resolution: 6.67 pixels mm^-1^	θ_max_ = 31.9°, θ_min_ = 4.2°
rotation method, ω scans	*h* = −14→13
Absorption correction: multi-scan (*LANA*; Koziskova *et al.*, 2016)	*k* = −13→13
*T*_min_ = 0.0002, *T*_max_ = 0.001	*l* = −7→7
6472 measured reflections	

### Hexafluoridouranium(VI) Refinement

**Table:**

Refinement on *F*^2^	Primary atom site location: dual
Least-squares matrix: full	*w* = 1/[σ^2^(*F*_o_^2^) + (0.0372*P*)^2^] where *P* = (*F*_o_^2^ + 2*F*_c_^2^)/3
*R*[*F*^2^ > 2σ(*F*^2^)] = 0.022	(Δ/σ)_max_ < 0.001
*wR*(*F*^2^) = 0.054	Δρ_max_ = 2.37 e Å^−^^3^
*S* = 1.03	Δρ_min_ = −1.68 e Å^−^^3^
784 reflections	Extinction correction: SHELXL-2019/2 (Sheldrick, 2015b), Fc^*^=kFc[1+0.001xFc^2^λ^3^/sin(2θ)]^-1/4^
38 parameters	Extinction coefficient: 0.0038 (4)
0 restraints	

### Hexafluoridouranium(VI) Special details

**Table:**

Geometry. All esds (except the esd in the dihedral angle between two l.s. planes) are estimated using the full covariance matrix. The cell esds are taken into account individually in the estimation of esds in distances, angles and torsion angles; correlations between esds in cell parameters are only used when they are defined by crystal symmetry. An approximate (isotropic) treatment of cell esds is used for estimating esds involving l.s. planes.

### Hexafluoridouranium(VI) Fractional atomic coordinates and isotropic or equivalent isotropic displacement parameters (Å^2^)

**Table:**

	*x*	*y*	*z*	*U*_iso_*/*U*_eq_	
U1	0.12887 (2)	0.250000	0.42275 (4)	0.01189 (11)	
F1	0.0110 (4)	0.250000	0.7420 (8)	0.0199 (9)	
F2	0.2469 (4)	0.250000	0.1039 (7)	0.0167 (8)	
F3	0.0096 (2)	0.0910 (3)	0.2627 (6)	0.0174 (5)	
F4	0.2471 (3)	0.0906 (4)	0.5800 (5)	0.0180 (6)	

### Hexafluoridouranium(VI) Atomic displacement parameters (Å^2^)

**Table:**

	*U*^11^	*U*^22^	*U*^33^	*U*^12^	*U*^13^	*U*^23^
U1	0.01144 (14)	0.01073 (13)	0.01350 (14)	0.000	−0.00002 (8)	0.000
F1	0.0201 (17)	0.022 (2)	0.018 (2)	0.000	0.0037 (16)	0.000
F2	0.0174 (16)	0.016 (2)	0.0164 (18)	0.000	0.0033 (14)	0.000
F3	0.0180 (11)	0.0131 (14)	0.0212 (13)	−0.0024 (11)	−0.0012 (11)	−0.0027 (11)
F4	0.0177 (11)	0.0158 (15)	0.0206 (14)	0.0025 (11)	−0.0004 (10)	−0.0010 (12)

### Hexafluoridouranium(VI) Geometric parameters (Å, º)

**Table:**

U1—U1^i^	5.1402 (4)	U1—F3	1.984 (3)
U1—U1^ii^	5.1072 (4)	U1—F3^iv^	1.984 (3)
U1—U1^iii^	5.0854 (4)	U1—F4	1.975 (3)
U1—F1	1.983 (4)	U1—F4^iv^	1.975 (3)
U1—F2	1.982 (4)		
			
F1—U1—F3^iv^	90.12 (11)	F4^iv^—U1—F2	89.92 (11)
F1—U1—F3	90.12 (11)	F4—U1—F2	89.92 (11)
F2—U1—F1	179.95 (14)	F4—U1—F3	90.20 (13)
F2—U1—F3	89.91 (11)	F4^iv^—U1—F3^iv^	90.20 (13)
F2—U1—F3^iv^	89.91 (11)	F4^iv^—U1—F3	179.56 (11)
F3^iv^—U1—F3	89.39 (17)	F4—U1—F3^iv^	179.56 (11)
F4^iv^—U1—F1	90.05 (11)	F4^iv^—U1—F4	90.21 (18)
F4—U1—F1	90.05 (11)		

Symmetry codes: (i) *x*−1/2, −*y*+1/2, −*z*+1/2; (ii) −*x*, *y*+1/2, −*z*+1; (iii) *x*, *y*, *z*+1; (iv) *x*, −*y*+1/2, *z*.
